# The Effect of the Image of Destinations on Household Income and Distribution: Evidence From China’s Tourist Cities

**DOI:** 10.3389/fpsyg.2022.859327

**Published:** 2022-04-21

**Authors:** Sheng Xu, Yunzhi Zhang, Jinghua Yin, Guan Huang

**Affiliations:** ^1^School of Health Management, Southern Medical University, Guangzhou, China; ^2^College of Economics, Jinan University, Guangzhou, China; ^3^Wenlan School of Business, Zhongnan University of Economics and Law, Wuhan, China

**Keywords:** destination image, tourism development, tourist motivation, urban planning policy, household income, income distribution, difference-in-differences

## Abstract

This paper examines the effect of the image of destinations on the wage income of resident households, and the corresponding income inequality, from a novel perspective. This work uses China’s excellent tourism city image program, which is an urban planning policy implemented by the central government across cities to enhance the image of the city destination in the minds of tourists, and then promote tourist motivation and local tourism development to assess the effect on household wage income and its distribution. Results show that the program significantly increases household wage income by increasing employment opportunities, promoting business and population agglomeration, and improving urban infrastructure. Additionally, the image of the city destination promotion causes an increase in income inequality among households.

## Introduction

Household income and its distribution are essential issues. Previous literature has explored the determinants of household income and its distribution from various perspectives (Bryan and Sevillasanz, 2011; Fisher et al., 2013; Eika et al., 2019). In the current paper, the determinants of wages and their distribution are investigated from the novel perspective of a city’s tourism development identified by the image of the city destination.

This paper contributes to the literature in three ways. First, existing studies focus on substantive policies such as land planning and tax changes (Sivadasan and Slemrod, 2008; Wei et al., 2017; Buettner et al., 2019; Huang et al., 2021), and few studies focus on the effect of tourism on income inequality at the regional level (Li et al., 2016; Lv, 2019; Chi, 2020). To our knowledge, this paper is among the first to use the destination image to identify tourism resources or tourism development and examine the effect of the destination image on income and income inequality at a household level. We use the China’s Excellent Tourism City Image (CETCI) program^1^ to identify tourism resources or tourism development. This paper complements the literature by providing evidence of the effect of urban planning policy (CETCI program) on household wage income.

Second, we propose a possible solution to the endogeneity problem to estimate the effect of tourism development on income and its distribution. Incera and Fernandez (2015) examined the effect of inbound tourism on income distribution in developed areas of Galicia, Spain, in 2008 using a social accounting matrix model. Li et al. (2016) examined the role of tourism development in reducing regional income inequality in China using a spatial temporal autoregressive model based on a conditional convergence framework. Alam and Paramati (2016) investigated the effect of tourism on income inequality using a fixed effect model with panel data from 49 developing economies. Nguyen et al. (2020) examined the combined influences of institutions and domestic/international tourism on the GINI index after tax and transfer in a global sample of 97 countries over the period between 2002 and 2014. The above literature provide important reference material for our study, but they do not fully address the endogeneity problem caused by the effect of tourism development on income distribution. Thus, we use the CETCI program to identify tourism development and solve the endogeneity problem using the difference-in-differences (DID) method.

Third, we find that in addition to increasing wage income and alleviating poverty, the CETCI program increases income inequality among households. This result is similar to that of Alam and Paramati (2016), who used GINI index estimation to measure income inequality, but we focus on household-level response. When estimating wage income inequality, focusing on the household level can help precisely estimate income dynamics. Li et al. (2016), Fang et al. (2020) and Nguyen et al. (2020) found that tourism development contributes significantly to the reduction of income inequality; however, we find contrary results. Our focus on household income can shed light on concerns about whether the effect of destination image on wage varies in accordance with household traits. We propose three alternative explanations for the channels underlying the empirical results. We extend Li et al. (2016), Fang et al. (2020), and Nguyen et al. (2020) by identifying the causal effect of the CETCI program. This work may motivate future research on the policies and channels linking the image of the destination and the wage income of households.

Neither the level nor the rate of change in the wage income of households before CETCI accreditation predicts when a city secures CETCI status. This finding suggests that the effect of CETCI accreditation is exogenous to the wage income of households. We use the DID method, examining exogenous cross-city, cross-year variation in the timing of CETCI accreditation to assess the causal effect of CETCI accreditation on the wage income of households. To tackle the identification challenge posed by omitted variables, we include household fixed effects and year fixed effects to control for all the time-invariant, unobserved characteristics. We also control for a set of time-varying variables on household and city characteristics.

The major findings are as follows: The image of city destination promotion has an overall positive effect on the wage income of households, and it causes an increase in wage income inequality across households. Generally, CETCI accreditation significantly increases the level of annual wage income of households by 2,400 RMB (345.4 USD, 13.6% relative to the average household wage income)^2^. The results are robust when controlling for time-varying household and city characteristics and household, city, and year-fixed effects. We show that accreditation significantly increases employment and business agglomeration and improves infrastructure, thus contributing to the annual wage income of households.

The remainder of this paper is organized as follows: Section “Literature Review and Background of CETCI Program” presents the literature review and institutional background of the CETCI program. Section “Data, Variables, and Estimation Strategy” explains the data, variables, and estimation strategy. Section “Results and Discussion” discusses empirical findings. Section “Discussion” concludes the study.

## Literature Review and Background of China’s Excellent Tourism City Image Program

### Literature Review

We aim to identify the effect of CETCI program on local household incomes. Tourism resources can promote tourist motivation and then the development of local tourism and economic development (Benkraiem et al., 2020; Li, 2020; Liu et al., 2021). Additionally, the image of city destination established by tourism resources, including civil and official recognition, plays an important role in regional development (Salehzadeh et al., 2016; Hwa-Kyung and Timothy, 2018). When a city has fruitful tourism resources, including natural and cultural landscapes, the resulting image enhances its ability to promote its external urban performance from a resource endowment standpoint (Su et al., 2018; Ebejer, 2019; Szubert et al., 2021). A good image can strengthen a city’s resource advantage and drive its development; such an image can be enhanced through government accreditation (Smith, 2006) and investment (Vanolo, 2015). Although existing literature has found that excellent tourism resources and intangible cultural heritage promote economic development (Hampton, 1998; Holzner, 2011; Joun and Kim, 2020), only a few studies have provided evidence on the effect of the tourism development on income distribution. Some literature suggests that tourism is a strategic factor that boosts the local economy of regions and countries and reduces regional income inequality (World Tourism Organisation, 2002; Li et al., 2016; Fang et al., 2020; Nguyen et al., 2020) and urban–rural income disparity (Shi et al., 2020). Other literature claims that high-income households benefit more than low-income ones, thus increasing income inequality (Incera and Fernandez, 2015; Alam and Paramati, 2016; Oviedo-García et al., 2018; Porto and Espinola, 2019).

We extend the previous mixed literature by investigating the causal effect of the CETCI program on household income and its distribution in China. CETCI is an accreditation granted by the Chinese government. It essentially recognizes a city’s tourism-relevant resources and tourism development.

### Background of China’s Excellent Tourism City Image Program

The CETCI program is an urban planning policy, which guides the city’s tourism industry planning by providing official publicity incentives to enhance the destination city’s image, competitiveness, and tourist motivation. The CETCI program can enhance the image of the city destination, tourist motivation, and then promote the development of local tourism. To standardize the management of tourism, maximize tourism resources, and promote the development of local tourism, the China National Tourism Administration launched the CETCI program in 1995^3^. The National Tourism Administration formulated “the inspection standards for China’s excellent tourist cities (for trial implementation)” and “the measures for the grant of China’s excellent tourist cities” in 1998. It bestows upon high-scoring cities the CETCI accreditation if they satisfy the selection criteria based on various comprehensive indicators. To secure CETCI, a city must go through three steps: application to the National Tourism Administration, inspection by the National Tourism Administration, and accreditation by the National Tourism Administration. Cities with tourism resources are encouraged to apply for CETCI accreditation. The CETCI publicizes destination cities based on the availability of tourism resources so it can attract numerous tourists and promote the development of urban tourism. In addition to its official publicity features, the CETCI program offers some substantive policies, such as land planning and tax reduction, that enhance tourism development.

In 2003, the National Tourism Administration revised its “Inspection Standards for China’s Excellent Tourist Cities” to standardize accreditation. As a result, we only focus on the cities selected for the CETCI list since 2003 to unify the criteria and reduce estimation error. The first list of 54 cities to win the title of CETCI was published in 1998, and the latest list was published in 2007. These cities can be divided into four types: (1) municipalities directly under the central government, (2) sub-provincial cities, (3) prefecture-level cities, and (4) county-level cities. This paper focuses on prefecture- and county-level cities granted CETCI accreditation. Municipalities and sub-provincial cities are excluded because of policy, institution, and economic agglomeration advantages that may interfere with our study. CETCI includes accreditation for prefecture- and county-level cities.^4^

From 2002 to 2006, the National Tourism Administration published the CETCI list four times^5^, and 132 prefecture- and county-level cities, covering 13 provinces, won the CETCI accreditation. Cities with CETCI accreditation have three ordinary features. First, they have unique tourism resources, such as natural scenery or cultural landscape. Second, CETCI accreditation plays an important role in introducing local tourism resources to tourists. Third, the CETCI program has some substantive policies such as land planning and tax reduction for tourism development. Based on these features, the CETCI program can bring several benefits, such as population and business agglomeration and infrastructure improvement, to attract tourists. Whether these benefits can ultimately be shared by households is the question that this paper attempts to answer.

## Data, Variables, and Estimation Strategy

In this section, we present data on the time of CETCI accreditation, household income, and household- and city- level characteristics. As an urban planning policy, we present the variables and the estimation strategy that we used for evaluating the effect of the CETCI program.

### Data and Variables

This paper focuses on the effect of CETCI accreditation on the wage income of households from accredited cities. Being granted CETCI status indicates that the city is outstanding in tourism—that is, the certified city has better tourism resources compared with other cities. Moreover, certified cities can better stimulate tourist motivation.

We use a large-scale dataset on household level to measure the annual wage income of households, [i.e., *Urban Household Survey* (UHS)].^6^ This dataset is an annual survey of 3500–4000 households across China and is conducted by the Urban Survey Team of the National Bureau of Statistics of China. The UHS is a representative sampling of the population, and some UHS samples trace households over time. The UHS provides information on the total annual wage income, savings, and consumption of households; income from other sources; and a wide array of demographic characteristics in the year prior to the survey. For our study, we limit the sample range to the years between 2002 and 2006 based on tracking rates. Data on annual wage income are used to quantify the direct outcomes of the CETCI program. Data related to average age, average education level, average number of working years of the household, and number of people with income in the household, are used as micro control variables.

We obtain a detailed list of the names of CETCI-certified cities from the official State Council document, “Interim Measures for the Administration of the Establishment of China’s Excellent Tourist Cities and Inspection Standards for China’s Excellent Tourist Cities.” During the sample period from 2002 to 2006, the National Tourism Administration published four annual lists.^7^ We set the general CETCI dummy variable to 1 if households from cities were granted CETCI status between 2002 and 2006; we set it to 0 if households from cities were not granted CETCI status before 2006. We define the treatment intensity parameter as 1 when households from the administrative area only have one CETCI accreditation. When households from the administrative area have two accreditations, including prefecture and county levels, the treatment intensity is defined as 2. According to the classification, 390 households and 914 households had been exposed to treatment intensity 2 and 1 by the end of 2006, respectively^8^.

Data on prefecture-level city characteristic variables are collected from *China City Statistical Yearbook* and *China Urban Construction Statistical Yearbook*. City-level variables, which include ratio of employees to the total population, ratio of tourists to the total population, population density, number of state-owned enterprises (SOEs) and non-state-owned enterprises (non-SOEs) with annual sales revenue of over RMB 5 million, and fixed asset investment, are used in the econometric model. The Section “Mechanism Analysis” uses ratio of employees to the total population, the ratio of leasing and commercial service personnel to employment, number of SOEs and non-SOEs with an annual sales revenue of more than RMB 5 million, ratio of self-employed people to the total population, fixed asset investment, and urban road area per capita.

To evaluate the effect of the CETCI program on the annual wage income of households, we construct a new panel dataset by integrating the data collected above. These data have four parts: the annual wage income of urban households, the CETCI accreditation list, the characteristics of urban households, and the characteristics of the city. Table 1 presents additional details on the construction of the variables used in our analysis. In this paper, households from cities that were granted CETCI status between 2002 and 2006 are included in the treatment group, and those that were not granted CETCI accreditation before 2006 are included in the control group.

**TABLE 1:** Descriptive statistics.

		Control group			Treatment group			Description
			
	Unit	Obs	Mean	*SD*	Obs	Mean	*SD*	
**Household income indicators**								
HWI	10^4^ Yuan	3965	1.626	1.375	4570	1.886	1.825	Total annual wage income of household
**Household characteristic variables**								
Average age of household	Years	3965	41.801	13.371	4570	43.725	13.708	Average age of household members
Average education level of household	Years	3965	4.832	1.157	4570	4.769	1.223	Average education level of household members
Average working years of household	Years	3965	18.740	11.722	4570	16.509	11.735	Average working years of household members
Number of people with income in the household	Persons	3965	24.711	7.514	4570	25.234	8.163	Number of household members with income (monthly average)
**Characteristics of prefecture-level cities**								
Ratio of employees to the total population		109	0.108	0.110	111	0.079	0.035	Employees divided by total population in prefecture-level cities
Ratio of tourists to the total population		109	9.910	4.987	111	19.148	37.245	Tourists received divided by total population in prefecture-level cities
Population density	Persons/km^2^	109	359.814	261.144	111	502.529	317.292	Total urban population divided by total urban area
Number of SOEs and non-SOEs with an annual sales revenue of more than RMB 5 million	Enterprises	109	3.253	2.126	111	7.665	9.079	Number of SOEs and non-SOEs with annual sales revenue of more than RMB 5 million in prefecture-level cities
Fixed asset investment	1 billion	109	12.193	11.989	111	19.813	19.014	Fixed asset investment in prefecture-level cities

*The treatment group includes households from cities granted CETCI accreditation. The control group consists of households from cities that were not granted CETCI status before 2006. Education level is a category variable in UHS. The measures of education level are as follows: (1) illiteracy, (2) informal education, (3) the highest level of education received is primary school, (4) the highest level of education received is junior high school, (5) the highest level of education received is ordinary high school or vocational high school, (6) the highest level of education received is secondary vocational school, (7) the highest level of education received is higher vocational education, (8) the highest level of education received is undergraduate, (9) the highest levels of education received is a master’s or doctor’s degree.*

### Specification

The CETCI program can be regarded as exogenous shock for two reasons. The tourism resources of cities can be considered exogenous because most tourism resources include historical and cultural heritage and natural landscapes. In addition, the wage income of households is not a criterion for the CETCI program; thus, it is not related to the accreditation. We use the DID method to assess the relation between CETCI accreditation and the annual wage income of households based on the following specification:


(1) $$W\,I_{h\,t}=\beta_{0}+\beta_{1}\,C\,E\,T\,C\,I_{h}\times a\,f\,t\,e\,r_{t}+\beta_{2}\,X_{h\,t}+\mu_{h}+\nu_{t}+\varepsilon_{h\,t}$$

In equation (1), the dependent variable *WI*_*ht*_ measures the annual wage income for household *h* in year *t*. The DID estimation approach enables us to control for omitted variables by including two sets of fixed effects in the model. μ_*h*_ denotes household fixed effects, capturing all the time-invariant, unobserved characteristics of households (e.g., gender, endowment, and location characteristics). ν_*t*_ represents year fixed effects, capturing all yearly factors common to all households (e.g., personal income tax, monetary and social security policy, national changes in regulations and laws, and long-term trends in income distribution). X_*ht*_ is a set of household- and city-level variables that captures the characteristics of a household and city that may directly or indirectly influence the annual wage income. ε_*ht*_ is the error term. *CETCI*_h_ equals 1 if a household comes from a city granted CETCI status between 2002 and 2006 and 0 otherwise. The dummy variable *after*_*t*_ indicates the post-treatment year. Hence, the variable of interest *CETCI*_h_×*after*_t_ equals 1 for household–year observations after a household from a city has obtained CETCI accreditation and 0 otherwise. To deal with potential heteroskedasticity and serial correlation, we cluster the standard errors at the household and city-year levels (two-way clustering). Therefore, coefficient β_1_ indicates the effect of CETCI accreditation on the annual wage income of households. A positive significant β_1_ suggests that the CETCI program has a positive effect on the annual wage income of households, whereas a negative significant β_1_ indicates that the CETCI program decreases the annual wage income of households.

## Results and Discussion

In this section, we present the empirical results, mechanisms, and heterogeneity analysis.

### Main Results

#### China’s Excellent Tourism City Image Program and Household Wage Income

The results of the effect of CETCI accreditation on wage income are reported in Table 2.^9^ Specifically, we assess the effect of CETCI accreditation on wage income using household wage income and three regression specifications. The wages of households are measured by total household annual wage income (HWI).

**TABLE 2:** Effect of CETCI accreditation on wage income.

	(1)	(2)	(3)
	HWI	HWI	HWI
CETCI × after	0.232***	0.150***	0.136**
	(0.079)	(0.056)	(0.060)
Average age of household		−0.039***	−0.039***
		(0.004)	(0.004)
Average level of education of household		0.479***	0.482***
		(0.033)	(0.033)
Average number of working years of household		0.021***	0.020***
		(0.004)	(0.004)
Number of people with income in household		0.042***	0.042***
		(0.004)	(0.004)
Ratio of employees to total population			2.805*
			(1.562)
Ratio of tourists to the total population			0.004
			(0.006)
Population density			0.000
			(0.000)
Number of SOEs and non-SOEs with annual sales revenue of more than RMB 5 million			0.031*
			(0.018)
Fixed asset investment			−0.005
			(0.005)
Year FE	Yes	Yes	Yes
Household FE	Yes	Yes	Yes
Observations	8535	8535	8535
*R*-squared	0.6175	0.7371	0.7381

Observations are at the household–year level. Column (1) has no other control variables. In column (2), we only control for numerous household-specific characteristics. In column (3), we control for numerous household- and city-specific characteristics. CETCI × after is an indicator variable that equals 1 if an observation from a city occurs after the CETCI program has started during the sample period and 0 otherwise. In columns (1)–(3), the dependent variable is HWI, which is reported in the Table 2 heading. All regressions control for year and household fixed effects. Standard errors are clustered at the household and city-year level (two-way clustering) and are enclosed in parentheses. *, **, and *** indicate statistical significance at the 10, 5, and 1% levels, respectively.

Columns (1)–(3) show the regression of presence and absence household and urban characteristic variables. Overall, Table 2 indicates that CETCI accreditation substantially increases the wage income of households. The coefficients of the treatment indicator *CETCI*_h_×*after*_t_ are positive and statistically significant at the 5% level in all three regressions in columns (1)–(3) of Table 2. For example, the results in column (1) suggest that CETCI accreditation induces a 2,400 RMB increase in the annual wage income of households.

Column (1) shows the estimated results, including household and year fixed effects, and column (3) adds numerous household- and city-specific characteristics. Table 2 indicates that CETCI accreditation in a city increases the wage income of households, even when controlling for several household- and city-level factors. Some of the control variables are highly related to the wage income of households. Such variables include household-level factors, such as the average age, average education level, and average number of working years of the household, and number of people with income in the household; and city-level factors, such as ratio of employees to the total population, number of SOEs and non-SOEs with an annual sales revenue of more than RMB 5 million, and fixed asset investment. The results of the effect of CETCI accreditation are robust to controlling for household and city characteristics. Overall, our empirical results confirm that tourism resources reduce poverty (Folarin and Adeniyi, 2020; Zhao, 2020; Zhao and Xia, 2020), but the effect on income distribution and inequality needs further study.

In Section “Validity and Robustness,” we conduct robustness tests, including validating the parallel trend assumption by investigating whether our findings persist for a placebo test; checking whether time-varying omitted variables bias the DID results; controlling confounding factors, such as the severe acute respiratory syndrome (SARS) epidemic and other contemporary city image programs; examining the effect of the CETCI program on annual wage income trends; and conducting a placebo test with the random assignment of CETCI accreditation in accordance with La Ferrara et al. (2012).

#### China’s Excellent Tourism City Image Program and Household Wage Income Inequality

Although the results in Table 2 demonstrate that the annual wage income of households in cities with CETCI accreditation is significantly higher than that in cities without CETCI accreditation, but the analyses have yet to provide information on whether the direction and magnitudes of income increase in accordance with the income level.

We address this issue by examining the effect of CETCI accreditation on the annual wage income of households across the full distribution of incomes. Specifically, we calculate the *p*th percentile of the annual wage income of each household *h* in a city in year t, WI(p)_ht_. We perform this for *p* = 10, 20, 30, 40, 50, 60, 70, 80, and 90. We then run nine regressions of the form


(2) $$W\,I\,{\left(p\right)}_{h\,t}=\theta_{0}+\theta_{1}\,C\,E\,T\,C\,I_{h}\times a\,f\,t\,e\,r_{t}+\theta_{2}\,X_{h\,t}+\mu_{h}+\nu_{t}+\varepsilon_{h\,t}$$

where the regressions are run for each *p*th percentile of the income distribution. Table 3 shows the estimated results θ_1_ from each of these nine regressions and indicates whether the estimates are significant at the 1% level.

**TABLE 3:** Effect of CETCI on different percentiles of annual wage income distribution.

	(1)	(2)	(3)	(4)	(5)	(6)	(7)	(8)	(9)
	p10	p20	p30	p40	p50	p60	p70	p80	p90
CETCI × after	0.001	−0.006	0.210**	0.229***	0.108	0.188*	0.280***	0.382***	0.447***
	(0.037)	(0.059)	(0.085)	(0.081)	(0.088)	(0.097)	(0.091)	(0.093)	(0.129)
Controls	Yes	Yes	Yes	Yes	Yes	Yes	Yes	Yes	Yes
Year FE	Yes	Yes	Yes	Yes	Yes	Yes	Yes	Yes	Yes
Household FE	Yes	Yes	Yes	Yes	Yes	Yes	Yes	Yes	Yes
Observations	8535	8535	8535	8535	8535	8535	8535	8535	8535
*R*-squared	0.8750	0.9224	0.9027	0.8954	0.8965	0.8811	0.8936	0.9304	0.9267

All observations are at the household–year level. CETCI × after is an indicator variable that equals 1 if an observation from a city occurs after the CETCI program has started during the sample period and 0 otherwise. All regressions control for year, household fixed effects, and numerous household- and city-specific characteristics. Standard errors are clustered at the household and city-year level (two-way clustering) and appear in parentheses. *, **, and *** indicate statistical significance at the 10, 5, and 1% levels, respectively.

Table 3 shows that CETCI accreditation improves the income of the high-income group more than that of the low-income group. Specifically, the CETCI coefficient increases substantially with the increase in income quantile (except for *p*50 and *p*60). Therefore, the overall conclusion is that high-wage income households benefit more than low-wage income ones, contributing to the increase in wage income inequality across households. The finding that the image of the city destination promotion increases wage income inequality among households is consistent with the results of studies showing that tourism consumption increases the disposable income inequality of households (Incera and Fernandez, 2015) and tourism industry increases income inequality in developing economies (Alam and Paramati, 2016; Oviedo-García et al., 2018; Porto and Espinola, 2019). However, a contrary view is that tourism can reduce regional income inequality in developing economies (Li et al., 2016; Fang et al., 2020). At least three explanations are offered for this. First, whether the tourism industry expands or shrinks income inequality depends on international tourism (Nguyen et al., 2020) or domestic tourism. Several studies (Bowden, 2005; Athanasopoulos and Hyndman, 2008) suggested that tourism spatial polarization, which is treated as enlarging the existing inequalities, is mainly correlated with international rather than domestic tourism. Li et al. (2016) mainly focused on the domestic tourism industry in China, whereas this paper focuses on tourism destination publicity, which includes domestic tourism and international tourism. Second, the GINI index of provincial per capita GDP is used to measure regional inequality in Li et al. (2016), whereas this paper focuses on the inequality of household wage income. Third, the reduction of regional income inequality may be triggered by economic development, but Li et al. (2016) does not completely solve this confusion factor.

### Validity and Robustness

In this section, we conduct robustness checks on the estimation results. We report the details of the estimation in Column 3 of Table 2.

#### Validating the Parallel Trend Assumption

**FIGURE 1:**
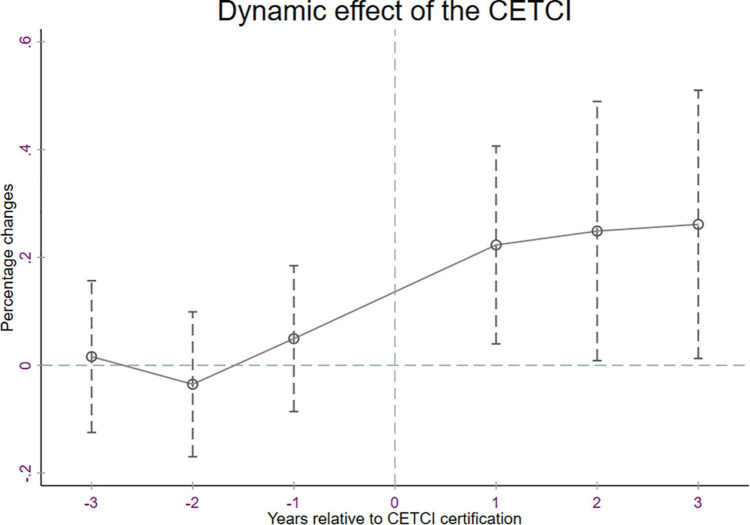
Dynamic effect of CETCI accreditation on the annual wage income of households. The horizontal axis measures the number of years since the CETCI accreditation was granted. The plots connected by the solid line indicate changes in household wage income compared with the period immediately before the CETCI accreditation. The dotted lines indicate 95% confidence intervals. Standard errors are clustered at the household and city-year level (two-way clustering).

DID estimation rests on the parallel trend assumption that no significant difference exists between the treatment and control groups in terms of the trend of the annual wage income of households before CETCI accreditation, that is, in a regression of the year of CETCI accreditation on the annual wage income of households before CETCI accreditation, the coefficients of *CETCI*_h_×*after*_t_ should be not significant. To test this hypothesis, we investigate placebo (non-existent) CETCI accreditation, which is acquired before a CETCI is granted to a household from a city. When the parallel trend hypothesis holds, we check the insignificant differential effects of these placebo CETCI accreditation on the treatment and control samples. Figure 1 presents the point estimate of the effect of CETCI accreditations on the annual wage income of households with 95% confidence bands. It shows that (1) placebo CETCI accreditations do not improve the annual wage income of households, (2) the effect of CETCI accreditations on the annual wage income of households rapidly materializes, and (3) the estimated effect of CETCI accreditations on the annual wage income of households weakens following its accreditation. The horizontal axis measures the number of years since the household from the city received a CETCI accreditation. The points connected by the solid line denote the coefficients of the CETCI dummy variables. These coefficients are not significantly different from 0 in every year before the CETCI status was granted, thus supporting the parallel trend hypothesis.

#### Difference-in-Difference-in-Differences Estimation

The results of DID estimation may be reasonably debatable because some time-varying city characteristics, such as level of economic development, may be related to the regressor of interest and thus bias the estimation. The annual wage income gap of households may have a gap due to varying economic development levels between the control and treatment groups. To eliminate such gaps, we conduct difference-in-difference-in-differences (DDD) estimation to check whether potentially omitted time-varying variables bias the DID results. We must find a new pair of control and experimental groups that are not affected by the CETCI program. We take advantage of the fact that CETCI accreditation is not affected by the level of urban economic development and expand the baseline specification with a triple interaction term, multiplied by dummy variables of *CETCI*_h_, *after*_*t*_, and *group*_*c*_. The DDD estimation specification according to Cai et al. (2016) is as follows:


(3) $$W\,I_{h\,t\,c}=\tau_{0}+\tau_{1}\,C\,E\,T\,C\,I_{h}\times a\,f\,t\,e\,r_{t}\times g\,r\,o\,u\,p_{c}+\tau_{2}\,X_{h\,t\,c}+\eta_{h\,t}+\phi_{h\,c}+\lambda_{t\,c}+\varepsilon_{h\,t\,c}$$

where *group*_*c*_ is a dummy variable that equals 1 when households come from a more developed city and 0 otherwise; *group*_*c*_ = 1if a city’s GDP in year *t* is greater than the median of the sample cities and 0 otherwise. The triple difference estimation technique allows us to control for full sets of household-city fixed effects, household -year fixed effects, and city-year fixed effects, in which all time-invariant, unobserved household and city characteristics that potentially shape the annual wage income across households are properly dealt with. All other controls are as previously defined. Therefore, the third difference^10^ compares the annual wage income of households from developed and underdeveloped cities, and τ_1_ captures the differential responses of the annual wage income of households to CETCI accreditation across cities with different economic development levels.

Columns (1)–(2) in Table 4 show that the coefficients of urban GDP and per capita GDP are not significant, Thus, CETCI accreditation is not affected by the level of urban economic development. The DDD estimation results are presented in columns (3)–(6) of Table 4. We find that the triple interaction term is consistently positive and statistically significant, indicating that our main results are not driven by potentially omitted time-varying variables and are robust.

**TABLE 4:** Difference-in-difference-in-differences (DDD) estimation results for the effect of CETCI accreditation on wage income.

	(1)	(2)	(3)	(4)	(5)	(6)
	CETCI × after	CETCI × after	HWI	HWI	HWI	HWI
CETCI × after × group			0.457***	0.268**	0.398***	0.222*
			(0.122)	(0.106)	(0.147)	(0.131)
Urban GDP	0.002					
	(0.001)					
Urban per capita GDP		0.022				
		(0.068)				
Controls	No	No	No	Yes	No	Yes
Household–year fixed effect	No	No	No	No	Yes	Yes
City–household fixed effect	No	No	No	No	Yes	Yes
City–year fixed effect	No	No	No	No	Yes	Yes
City FE	Yes	Yes	No	No	No	No
Year FE	Yes	Yes	Yes	Yes	No	No
Household FE	No	No	Yes	Yes	No	No
Observations	230	230	8535	8535	8535	8535
*R*-squared	0.6590	0.6576	0.6187	0.7385	0.9215	0.9463

Observations in columns (1)–(2) are at the city–year level. Observations in columns (3)–(6) are at the household–year level. The CETCI × after × group indicator equals 1 during sample years in which a household from a more developed city is granted CETCI status. Columns (3), (5) and (4), (6) show the regression of presence and absence control variables, respectively. Standard errors are clustered at the city level (one-way clustering) in Columns (1)–(2) and at the household and city-year level (two-way clustering) in Columns (3)–(6) and are enclosed in parentheses. *, **, and *** indicate statistical significance at the 10, 5, and 1% levels, respectively.

#### Post-china’s Excellent Tourism City Image Trend

The point estimates in the event study (Jacobson et al., 1992; Autor et al., 2014) suggest that the CETCI program affects not only the level but also the trends in the annual wage income of households. To this end, we modify Equation (1) to examine the effect of the CETCI program on the annual wage income trends. Specifically, we define the post-CETCI trend as *F*_*ht*_ = *t*−*c*_*h*_ if *t*≥*c*_*h*_ and 0 otherwise, where C_h_ denotes the year in which households from the city were granted CETCI accreditation. The specifications are as follows:


(4) $$W\,I_{h\,t}=\alpha_{0}+\alpha_{1}\,C\,E\,T\,C\,I_{h}\times a\,f\,t\,e\,r_{t}+\alpha_{2}\,X_{h\,t}+\alpha_{3}\,F_{h\,t}+\mu_{h}+\nu_{t}+\varepsilon_{h\,t}$$

where *F*_*ht*_ is the trend variable that represents how long the CETCI status has been in effect. As defined in Section “Specification,” *CETCI*_h_×*after*_t_ is the interest variable indicating the CETCI program; hence, the effect of the CETCI program on the level of the outcome variables *WI*_*ht*_ is denoted by α_1_, whereas α_3_ measures the effect of the CETCI experiment duration on the outcome variables. All other variables are as previously defined. Table 5 presents the results, with columns (1)–(2) providing estimates of Equation (4). Columns (3)–(4) are re-estimations of Equation (4) after taking the logarithm of HWI. The coefficient of trend variable *F*_*ht*_ is positive but statistically insignificant in all columns, suggesting that the durability of CETCI accreditation does not affect the annual wage income of households. The coefficients of *CETCI*_h_×*after*_t_ for all dependent variables are consistent with those previously reported in Table 2, indicating that our main results are robust in this test.

**TABLE 5:** Effect of CETCI accreditation on wage income.

	(1)	(2)	(3)	(4)
	HWI	HWI	lnHWI	lnHWI
CETCI × after	0.214***	0.141**	0.069**	0.043**
	(0.082)	(0.062)	(0.030)	(0.020)
*F*	0.041	−0.014	−0.005	−0.013
	(0.049)	(0.039)	(0.015)	(0.010)
Controls	No	Yes	No	Yes
Year FE	Yes	Yes	Yes	Yes
Household FE	Yes	Yes	Yes	Yes
Observations	8535	8535	8535	8535
*R*-squared	0.6176	0.7381	0.6321	0.7891

All observations are at the household–year level. CETCI × after is an indicator variable that equals 1 if an observation from a city occurs after the CETCI program has started during the sample period and 0 otherwise. F is an indicator variable that denotes a linear trend after the commencement of the CETCI program. In columns (3)–(4), the dependent variable is lnHWI, which is a natural logarithm of HWI. Columns (1), (3) and (2), (4) show the regression of presence and absence control variables, respectively. All regressions control for year and household fixed effects. Standard errors are clustered at the household and city-year level (two-way clustering) and are given in parentheses. *, **, and *** indicate statistical significance at the 10, 5, and 1% levels, respectively.

#### Other Contemporary Events

If other events happen simultaneously with CETCI accreditation in whole or in part, any finding about CETCI treatment effect is affected. These confounding factors may also provide alternative explanations for our findings. For this reason, in this section, we investigate whether the presence of confounding factors, including an exogenous shock and other contemporary city image programs, can alter the previous findings.

An important exogenous shock that occurred during the sample period is the SARS^11^ outbreak; this event may have caused differences in the annual wage income of households between the treatment and control groups because of differences in the severity of the outbreak between the two groups. Lockdowns and shutdowns triggered by the epidemic can affect household wages (Ceylan and Ozkan, 2020). Therefore, the 2003 SARS event may have introduced potential bias to our previous findings. To address this concern, the benchmark Equation (1) is augmented with *SARS*_*ht*_, where *SARS*_*ht*_ is a dummy variable that equals 1 for households that may have been affected by SARS in 2003 and 0 otherwise. We collect the number of confirmed SARS cases in each province in 2003. If the number of confirmed cases in the province where the household are located is greater than or equal to 1 and the sample year is 2003, then *SARS*_*ht*_ is equal to 1; otherwise, it is equal to 0. The reason for this design is that whether a province has a confirmed case affects whether it initiates a public health event response mechanism and response level. The response level involves the intensity of lockdowns and shutdowns.

The results in columns (1)–(2) of Table 6 show that the coefficients on *CETCI*_h_×*after*_t_ are consistent with previously reported ones. These results are attributed to changes in the annual wages of households to CETCI accreditation instead of the SARS outbreak in 2003. Further, column (1) of Table 6 shows that the coefficient of the *SARS*_*ht*_ variable is significantly negative, indicating that SARS reduces the annual wage income of households without control variables. However, the significance level of *CETCI*_h_×*after*_t_ is not affected by the presence or absence of control variables, indicating that our results are robust.

**TABLE 6:** Robustness to controlling for possible confounders to wage income.

	(1)	(2)	(3)	(4)
	HWI	HWI	HWI	HWI
CETCI × after	0.231***	0.136**	0.233***	0.129**
	(0.078)	(0.060)	(0.078)	(0.059)
SARS	−0.151*	−0.067		
	(0.080)	(0.059)		
Other_image			0.055	0.010
			(0.091)	(0.067)
Controls	No	Yes	No	Yes
Year FE	Yes	Yes	Yes	Yes
Household FE	Yes	Yes	Yes	Yes
Observations	8535	8535	8535	8535
*R*-squared	0.6177	0.7382	0.6175	0.7381

All observations are at the household–year level. CETCI × after is an indicator variable that equals 1 if an observation from a city is after the CETCI program starts and 0 otherwise. SARS is an indicator variable that equals 1 if a province had one or more confirmed SARS cases in 2003. Other_image is an indicator variable that equals 1 if an observation from a city occurs after the other city image program has started during the sample period and 0 otherwise. Columns (1), (3) and (2), (4) show the regression of presence and absence control variables, respectively. All regressions control for year and household fixed effects. Standard errors are clustered at the household and city-year level (two-way clustering) and are given in parentheses. *, **, and *** indicate statistical significance at the 10, 5, and 1% levels, respectively.

Another potential confusion factor is other contemporary city image programs. Similar to the CETCI program, the Chinese government or authoritative media have implemented several programs aimed at improving the image of various cities. These city image programs, which were implemented in the post-CETCI period, could have triggered the different behavior of the treated and control groups and may even have a consistent influence mechanism with the CETCI program. To verify this, we investigate whether other contemporary programs (policies), including the national civilized city image program, the national health city image program, the national garden city image program, the national historical and cultural city image program, and China’s charming city image program, can confound our results.

Similar to the exclusion of SARS confusion, the benchmark model is augmented with *other*_*image*_*ht*_, where *other*_*image*_*ht*_ is a dummy variable that equals 1 for household–year observations after households from cities that launched the other contemporary city image programs mentioned above between 2002 and 2006, and 0 otherwise. The results are reported in columns (3)–(4) of Table 6. We find a similar estimate in this new model in terms of statistical significance and magnitude, indicating that our findings are not driven by other city images.

In summary, these results rule out the alternative explanation stated above.

#### Placebo Test

Our results may be biased because of the omitted variables at the city–household level, that is, CETCI accreditation is endogenous relative to the annual wage income of households if income factors are included directly or indirectly in the government’s criteria for granting CETCI status. To check the exogenous assumption of CETCI accreditation further, we take a closer look at identification issues in this paper.

**FIGURE 2:**
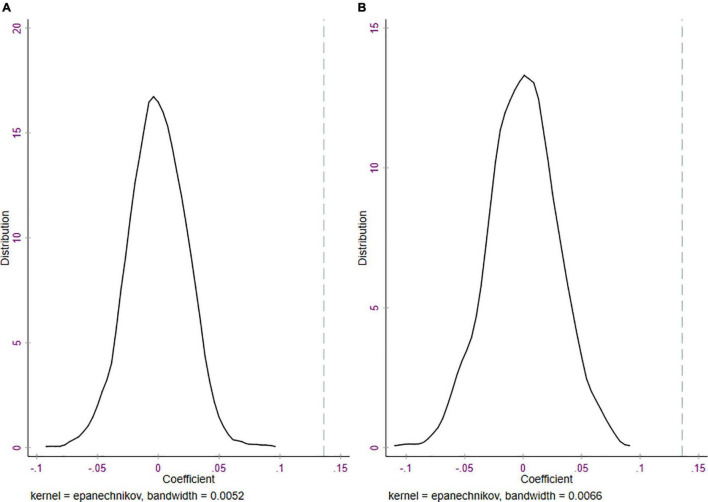
**(A)** Distribution of estimated coefficients with placebo households from cities and CETCI accreditation years. **(B)** Distribution of estimated coefficients with placebo cities and CETCI accreditation years. The dotted lines indicate the actual coefficient of the CETCI accreditation dummy variable.

Specifically, we conduct two placebo tests. The first placebo test is conducted by randomly generating a year of CETCI accreditation during the sample period (2002–2006) and assigning household samples to the treatment group; for similar practices, see (Chetty et al., 2009; La Ferrara et al., 2012; Liu and Zhang, 2019). The second placebo test is conducted by randomly generating a year of CETCI accreditation during the sample period (2002–2006) and assigning a city to the treatment group. As a result, the sample of households from the placebo city is included in the treatment group. From our random draws, we construct two false CETCI^false^ dummy variables. We then re-estimate our benchmark model, Equation (1), using the false CETCI^false^ dummy variable and store the estimates. If the CETCI accreditation is exogenous, then we find that the coefficient $\beta_{1}^{f\,a\,l\,s\,e}$ of the variable of interest CETCI^false^ should be close to 0 and insignificant. In other words, a randomly constructed CETCI^false^ program should have no effect on the annual wage income of households. To avoid the interference of rare events, we repeat this random data-generating process 1,000 times. The densities of the estimated coefficients on CETCI^false^ for the first and second placebo tests are shown in Figures 2A,B. The distribution of the estimated coefficients on two placebo CETCI^false^ variables are all centered around 0, as expected. Moreover, our estimate using the true CETCI accreditation year and sites from column (3) of Table 2 [i.e., 0.136 as indicated by the dotted lines in Figures 2A,B] clearly lies outside the range of the coefficients estimated in two placebo tests, suggesting that our findings are not severely biased by the omitted variables.

### Mechanism Analysis

In the previous sections, we identified a significant positive effect of CETCI accreditation on the annual wage income of households. In this subsection, we provide some further evidence to shed light on the underlying mechanisms through which CETCI accreditation may affect the wage income.

Three potential mechanisms, employment, business, and infrastructure construction can be used to explain the effect of CETCI accreditation on the annual wage income of households. First, tourism creates employment in tertiary industries (Hughes, 1982; Ke et al., 2011). Compared with non-accredited cities, CETCI accreditation can attract numerous tourists and create additional jobs. The higher the employment rate is, the higher the wage income. Second, CETCI-accredited cities have the effect of people gathering because of their enhanced tourism motivation, thus promoting numerous business opportunities (Jaafar et al., 2011; Snyman, 2017). The development of businesses can increase HWI. Third, excellent tourism resources motivate the government to invest in large-scale infrastructure construction (Fallon and Kriwoken, 2003; Laslo and David, 2003; Lim et al., 2019), such as the construction of tourism routes and public network facilities. Infrastructure construction can increase the operational efficiency of enterprises and hence increase wage levels.

To test these three channels formally, we conduct two exercises. First, we regress the DID indicator on employment, business, and infrastructure. Second, we regress employment, business, infrastructure, and the DID indicator on the annual wage income of households. The employment creation effect is measured by the ratio of employees to the total population and the ratio of leasing employees to the total employees. The number of SOEs and non-SOEs with annual sales revenue of more than 5 million and the ratio of self-employed to the total population represent the business agglomeration effect. Fixed asset investment and per capita road area are used to measure the effect of infrastructure investment.

Table 7 shows the mechanism test results of the annual wage income of households. Column (1) shows the benchmark regression result. Columns (2)–(5) show that CETCI accreditation creates employment, especially in the leasing industry, and can improve the annual wage income of urban households. Columns (6)–(9) investigate the business agglomeration because of CETCI accreditation. This potential mechanism is also supported by our empirical findings, suggesting that the CETCI program improves the annual wage income of households by attracting large enterprises and promoting the development of self-employed businesses. We find a significant infrastructure investment effect associated with CETCI accreditation, as suggested by columns (10) and (12). The coefficient of *infrastructure_invest* in column (11) and the coefficient of *infrastructure_road* in column (13) are positive at the 10% level, suggesting that increased investment in infrastructure is an important channel through which CETCI accreditation affects the wage income of urban households and individuals.

**TABLE 7:** Effect of CETCI accreditation on the annual wage income of households: mechanism analysis.

	(1)	(2)	(3)	(4)	(5)	(6)	(7)	(8)	(9)	(10)	(11)	(12)	(13)
	HWI	Employment_e	HWI	Employment_l	HWI	Business_500	HWI	Business_self-employed	HWI	Infrastructure_invest	HWI	Infrastructure_road	HWI
CETCI × after	0.150***	0.002	0.142**	0.005***	0.141**	2.331*	0.112**	0.002***	0.144**	10.728**	0.109*	2.196***	0.136**
	(0.056)	(0.002)	(0.056)	(0.000)	(0.056)	(1.292)	(0.056)	(0.000)	(0.056)	(4.785)	(0.059)	(0.811)	(0.056)
Employment_e			4.014**										
			(1.646)										
Employment_l					1.955								
					(1.324)								
Business_500							0.016**						
							(0.007)						
Business_self-employed									2.994***				
									(1.010)				
Infrastructure_invest											0.004*		
											(0.002)		
Infrastructure_road													0.006***
													(0.002)
Controls	Yes	No	Yes	No	Yes	No	Yes	No	Yes	No	Yes	No	Yes
Year FE	Yes	Yes	Yes	Yes	Yes	Yes	Yes	Yes	Yes	Yes	Yes	Yes	Yes
Household FE	Yes	Yes	Yes	Yes	Yes	Yes	Yes	Yes	Yes	Yes	Yes	Yes	Yes
Observations	8535	8535	8535	8535	8535	8535	8535	8535	8535	8535	8535	8485	8485
*R*-squared	0.7371	0.9871	0.7374	0.7352	0.7373	0.9204	0.7379	0.8004	0.7375	0.8424	0.7376	0.5500	0.7376

Observations are at the household–year level. Column (1) is the benchmark regression result. CETCI × after is an indicator variable that equals 1 if an observation from the city occurs after the CETCI program has started and 0 otherwise. The measures of employment are as follows: (1) the ratio of employees to the total population (represented by employment_e in Table 6) and (2) the ratio of leasing employees to the total employees (represented by employment_l in Table 6). The measures of business are as follows: (1) the number of SOEs and non-SOEs with an annual sales revenue of more than 5 million (represented by business_500 in Table 6), (2) the ratio of self-employed to the total population (represented by business_self-employed in Table 6). The measures of infrastructure are as follows: (1) fixed asset investment (represented by infrastructure_invest in Table 6) and (2) per capita road area (represented by infrastructure_road in Table 6). In columns (1), (3), (5), (7), (9), (11), and (13), the dependent variables are total HWI, which is reported in the column heading. Standard errors are clustered at the household and city-year level (two-way clustering) and are enclosed in parentheses. *, **, and *** indicate statistical significance at the 10, 5, and 1% levels, respectively.

### Heterogeneous Effects

If CETCI accreditation drives the previous results, then it may have heterogeneous effects on treatment variables. To examine such heterogeneity, we use the full richness of the CETCI program.

#### Heterogeneous Effects on Different Control and Treatment Groups

Ideally, the effect of CETCI accreditation on the annual wage income of households is statistically significant in two DID regressions: dataset presence and absence in non-CETCI observations.

To test this hypothesis, we evaluate the effect of CETCI accreditation by excluding the sample of households from cities that were not accredited until 2006. Cities that have never been selected for the CETCI list may have poorer tourism resources compared with those selected for the list, that is, accredited cities have the same tourism resources, but differences occur in the time of obtaining CETCI accreditation. Therefore, the coefficient of *CETCI*_h_×*after*_t_ measures the official effect of CETCI accreditation (excluding tourism resource effects).

Table 8 estimates the official effect of CETCI accreditation. When examining CETCI accreditation and the annual wage income of households, we examine all samples, including those that are certified and those that are not. We are concerned only about the official publicity effect and exclude the tourism resource effects. Columns (1)–(4) present the results of the annual wage income of household related outcomes. Columns (1)–(2) are the regression results with non-certified observations, whereas columns (3)–(4) are the regression results without non-certified observations. The coefficients of the *CETCI*_h_×*after*_t_ indicators are all positive in columns (3)–(4), and the magnitudes are similar to those in columns (1)–(2). These results confirm the significant effect of official CETCI accreditation on the annual wage income of households.

**TABLE 8:** Effect of CETCI accreditation on wage income: without non-CETCI samples.

	(1)	(2)	(3)	(4)
	HWI	HWI	HWI	HWI
CETCI × after	0.232***	0.136**	0.231**	0.180**
	(0.079)	(0.060)	(0.112)	(0.086)
Controls	No	Yes	No	Yes
Year FE	Yes	Yes	Yes	Yes
Household FE	Yes	Yes	Yes	Yes
Observations	8535	8535	4570	4570
*R*-squared	0.6175	0.7381	0.5332	0.6841

All observations are at the household–year level. CETCI × after is an indicator variable that equals 1 if an observation from a city occurs after the CETCI program has started during the sample period and 0 otherwise. Columns (1), (3) and (2), (4) show the regression of presence and absence control variables, respectively. All regressions control for year and household fixed effects. Standard errors are clustered at the household and city-year level (two-way clustering) and appear in parentheses. *, **, and *** indicate statistical significance at the 10, 5, and 1% levels, respectively.

#### Single Versus Multiple Treatment Intensities

Households from different prefecture-level administrative areas have multiple treatment intensities (CETCI accreditation for prefecture and county levels). We define the treatment intensity as 1 when households from prefecture-level administrative areas have only one CETCI accreditation. We define the treatment intensity as 2 when households from prefecture-level administrative areas have two accreditations, including prefecture and county levels. We then assess whether the effect of the CETCI program is heterogeneous across different treatment intensities using the following panel data specification:


(5) $$W\,I_{h\,t}=\gamma_{0}+\gamma_{1}\,D_{h\,t}^{m\,u\,t\,i\,p\,l\,e}+\gamma_{2}\,F_{h\,t}^{m\,u\,t\,i\,p\,l\,e}+\mu_{h}+\nu_{t}+\varepsilon_{h\,t}$$

where $D_{h\,t}^{m\,u\,t\,i\,p\,l\,e}$ equals 1 if households from prefecture-level administrative areas have been exposed to treatment intensity 2 by 2006 and 0 otherwise. $F_{h\,t}^{m\,u\,t\,i\,p\,l\,e}$ is the trend variable that represents the duration of treatment intensity 2. All other controls are as previously defined. An important feature of this empirical setting is that households from various prefecture-level administrative areas could be exposed to different treatment intensities. Thus, estimating γ_1_ and γ_2_ coefficients using the intensity variation and time variation of treatments across prefecture-level administrative areas becomes feasible.

The results in Table 9 indicate the heterogeneous effect of treatment intensities on the wage income of households. Specifically, a positive treatment intensity effect is observed when moving from a single CETCI treatment (treatment intensity equals in columns [1]–[2]) to a dual CETCI treatment (treatment intensity equals 2 in columns [3]–[4]). These results confirm that treatment intensity effects increase in absolute magnitude when moving from the first to the second intensity. Moving from the first to the second treatment intensity in column (4), γ_1_ of the household regression group implies a level effect size of 0.255. No significant difference is observed in treatment trends across the intensities.

**TABLE 9:** Effect of CETCI intensity on wage income.

	(1)	(2)	(3)	(4)
	HWI	HWI	HWI	HWI
CETCI × after	0.232***	0.214***		
	(0.079)	(0.082)		
*F*		0.041		
		(0.049)		
$D_{h\,i\,t}^{m\,u\,t\,i\,p\,l\,e}$			0.233*	0.255*
			(0.125)	(0.133)
$F_{h\,i\,t}^{m\,u\,t\,i\,p\,l\,e}$				0.040
				(0.064)
Year FE	Yes	Yes	Yes	Yes
Household FE	Yes	Yes	Yes	Yes
Observations	8535	8535	8535	8535
*R*-squared	0.6175	0.6176	0.6169	0.6170

All observations are at the household–year level. CETCI × after is an indicator variable that equals 1 if an observation from a city occurs after the CETCI program has started and 0 otherwise. All regressions control for year and household fixed effects. Standard errors are clustered at the household and city-year level (two-way clustering) and are given in parentheses. *, **, and *** indicate statistical significance at the 10, 5, and 1% levels, respectively.

## Discussion

This paper examines the effect of the city destination image promotion on the wage income of resident households, and the corresponding income inequality. Specifically, we consider CETCI accreditation exogenous to households and use the CETCI program, which is an urban planning policy conducted by the China National Tourism Administration to enhance the image of the city destination in the minds of tourists and then promote tourist motivation and local tourism development, to evaluate the effect of CETCI accreditation on the annual wage income of households. In this section, we strengthen the depth of the interpretation of the results and, clarify the psychological significance. In the last two subsections, we discuss the policy implications and research caveats as well as future research directions.

### Conclusion

This paper extends the literature by estimating the causal effect of CETCI accreditation on the income of households. We use CETCI with the timing and regional variations generated by the program to check its causal effect. Our results indicate that the image of the city destination promotion contributes to the wage increase in households from a city with CETCI accreditation and to the increase in wage income inequality across households. In terms of different income percentiles, CETCI accreditation affects high-income groups more than low-income groups.

Evidence shows that CETCI accreditation increases the annual wage income of households by boosting job opportunities and providing a business-friendly environment. Specifically, we show that the employment rate, the number of SOEs and non-SOEs with an annual sales income of more than RMB 5 million, and the per capita road area increase significantly after a city obtains CETCI accreditation. Additionally, we find some heterogeneity of the effect depending on tourism resources. The effect of CETCI accreditation on wage income is larger in cities with higher accreditation intensity. This result implies that cities with substantial tourism resources and urban households can obtain great benefits.

### Psychological Significance

Distinctive brands play an important role in stimulating consumers’ consumption psychology. The psychological significance of this paper is to show that the official image of a tourist destination (similar to brands) can induce changes in other economic variables through the channel that stimulates tourists’ motivation, which inspires policymakers to consider using tourists’ consumption psychology to develop distinctive tourism industry and tourism destination image (brands).

### Policy Implications

The policy implications of our findings are as follows. On the one hand, the tourism industry is recognized as an effective means of alleviating poverty in developing economies (Oviedo-García et al., 2018). Our findings suggest that the promotion of tourism destination image accelerates the flow of tourism resource dividends to households. This paper not only adds to the evidence that tourism development can alleviate poverty but also inspires ways to alleviate poverty through tourism. The CETCI program can be used as an effective means of alleviate poverty. The government can use this program to prompt cities to integrate tourism resources and promote tourism development, thereby increasing residents’ income.

On the other hand, the adverse effects of the program on income inequality should not be ignored. The government can narrow the income inequality caused by tourism development by raising the minimum labor wage level in the tourism industry and providing job training for low-skilled tourism employees.

### Caveats and Future Research Direction

This paper provides an insight into how households gain from the place-based urban planning policy. However, the positive effects should be interpreted with caution because they might be closely linked to Chinese institutions. Further investigation is necessary to explore and compare the effects of the image of destinations in other relevant countries.

## Data Availability Statement

The raw data supporting the conclusions of this article will be made available by the authors, without undue reservation.

## Author Contributions

All authors listed have made a substantial, direct, and intellectual contribution to the work, and approved it for publication.

## Conflict of Interest

The authors declare that the research was conducted in the absence of any commercial or financial relationships that could be construed as a potential conflict of interest.

## Publisher’s Note

All claims expressed in this article are solely those of the authors and do not necessarily represent those of their affiliated organizations, or those of the publisher, the editors and the reviewers. Any product that may be evaluated in this article, or claim that may be made by its manufacturer, is not guaranteed or endorsed by the publisher.

## Footnotes

1 The CETCI program implemented by the Chinese government is an official accreditation of quality endorsement for the image of a tourist city destination. CETCI is an accreditation granted by the Chinese government. It essentially recognizes a city’s tourism-relevant resources and tourism development.

2 The average household wage income for all observations is 17,652 RMB (2539.9 USD).

3 In March 1995, the National Tourism Administration issued “the notice on the activities of creating and selecting excellent tourist cities in China.”

4 In China, a prefecture-level administrative area may include a prefecture-level city and several county-level cities.

5 The first CETCI list was released in 2003 and had 32 prefecture-level cities, such as Langfang (in Hebei Province), and 13 county-level cities, such as Yongji (in Shanxi Province). More details about the city name are available in Appendix Table 1.

6 In the UHS dataset, household income includes wage income, business income, and property income. We confirm the results of this paper by using the wage income because other sources of income account for a small proportion of the total income, and only the wage income has a significant effect. Thus, we focus on wage income rather than other sources of income throughout this paper.

7 A total of 73 prefecture-level cities and 59 county-level cities in China were included in the CETCI list from 2002 to 2006.

8 The constructed dataset comprises 1,707 households, with a sample period from 2002 to 2006.

9 Substantially consistent results are found at the individual level. To save space, the results for individual level are not reported in this paper.

10 Specifically, the first difference comes from the comparison of the household wage income in CETCI and non-CETCI cities (with the former CETCI accredited). The second difference compares the household wage income in more developed and underdeveloped cities (with the former having the effect of resource agglomeration). The last difference is the time of policy implementation, which divides the sample into pre- and post-treatment periods.

11 SARS was a global epidemic of severe acute respiratory syndrome (SARS) that emerged in Guangdong, China, in the end of 2002 and spread to Southeast Asia and throughout the world. It was not until mid-2003 that the epidemic was gradually eliminated. According to the official data released by National Health Commission of China, as of August 16, 2003, 5,327 cases of SARS had been reported in mainland China. The number of cases varied significantly across provinces, with Guangdong and Beijing the worst affected.
