# Supplementary material for: The Effect of the Image of Destinations on Household Income and Distribution: Evidence From China’s Tourist Cities
Source: Front Psychol. 2022 Apr 21;13:859327. doi: 10.3389/fpsyg.2022.859327 (PMC9069098; doi:10.3389/fpsyg.2022.859327)
Supplement: Supplementary file 1 [file Table_1.DOCX]

Supplementary Material

# Appendix A

**Table A1.** Cities with CETCI accreditation

| **Year of certification** | **List of prefecture-level cities** | **List of county-level cities** |
| --- | --- | --- |
| 2003 | Langfang, Baoding, Huhehaote, Hulunbeier, Huludao, Qiqihaer, Nantong, Lianyungang, Huzhou, Jiaxing,  Zhangzhou, Ganzhou, Weifang, Liaocheng, Rizhao, Sanmenxia, Anyang, Jiaozuo, Hebi, Xiangyang, Jinmen, e’zhou, Chenzhou, Dongguan, Chaozhou, Zhanjiang, Heyuan, Mianyang, Guangan, Zigong, Yan’an, Tianshui | Yongji, Manzhouli, Zhalantun, Linhai, Wenlin, Rushan, Lingbao, Xinzheng, Chibi, Kaiping, Qionghai, Geermu, Kashi |
| 2004 | Handan, Chifeng, Liaoyang, Linyi, Jining, Xuchang, Lanzhou, Xining, Kelamayi | a’er’shan, Xingcheng, Jiaohe, Ji’an, Tieli, Suyang, Fuyang, Haining, Zixing, Guiping, Langzhong, Kaili, Luxi, Hami |
| 2005 | Jincheng, Tieling, Panjin, Yancheng, Huaian, Quzhou, Zhoushan, Yingtan, Jingdezheng, Xinxiang, Shangqiu, Nanyang, Meizhou, Maoming, Yibin, Luzhou, Panzhihua, Ya’an, Lijiang, Zunyi, Zhangye, Wuwei | Huolinguole, Hulin, Zhangjiagang, Taicang, Rugao, Ruian, Lanxi, Fenghua, Zoucheng, Shouguang, Yuzhou, Changge, Wugang, Liuyang, Jiangyou, a’kesu, Yining, a’letai, Shihezi |
| 2006 | Shangrao, Chaoyang, Yingkou, Taizhou, Tonglin, Chizhou, Pingdingshan, Zhuzhou, Pingliang, Nanchong | Wu’an, Jintan, Dongtai, Jiangshan, Longkou, Haiyang, Zhangqiu, Huaying, Qionglai, Xichang, Hancheng, Bole, Changji |
